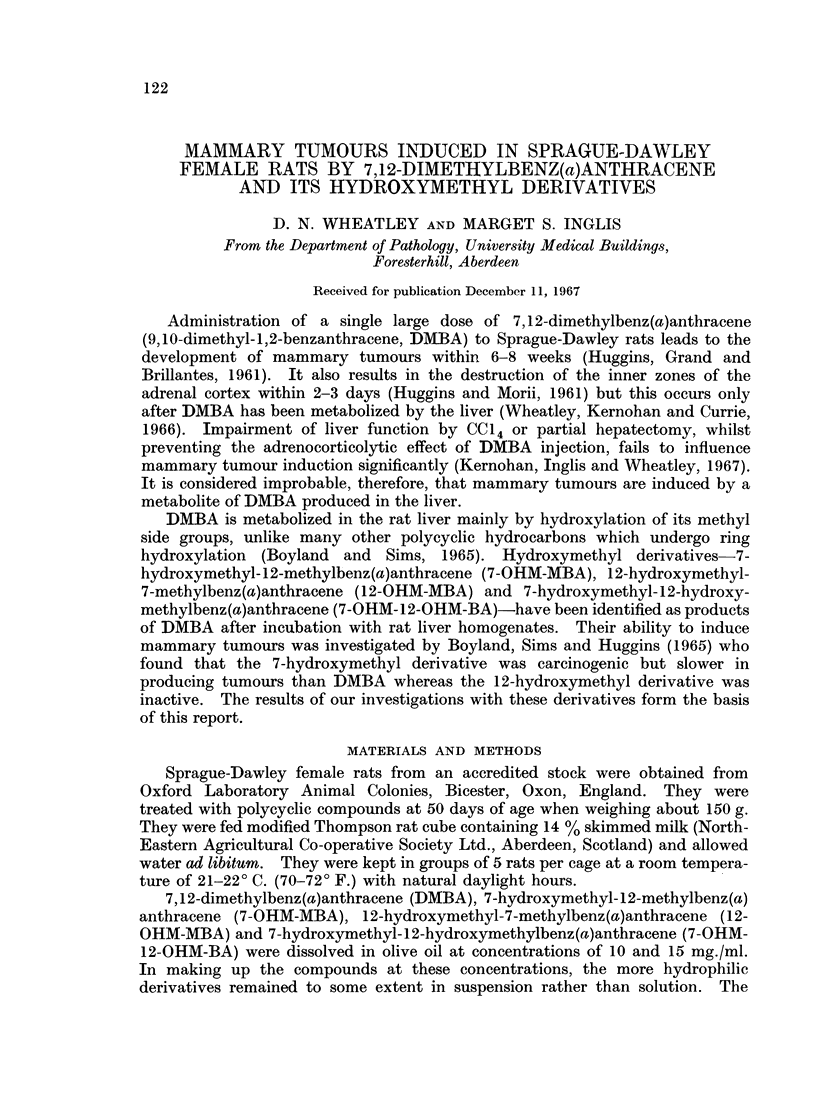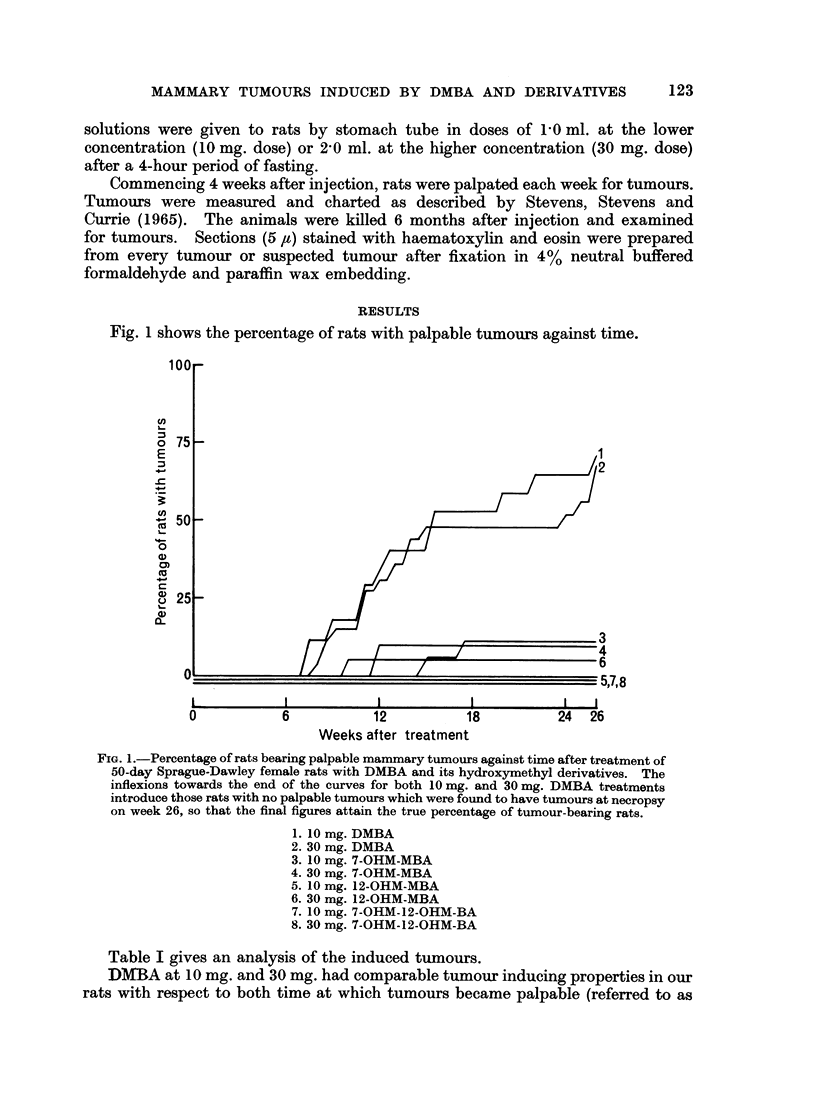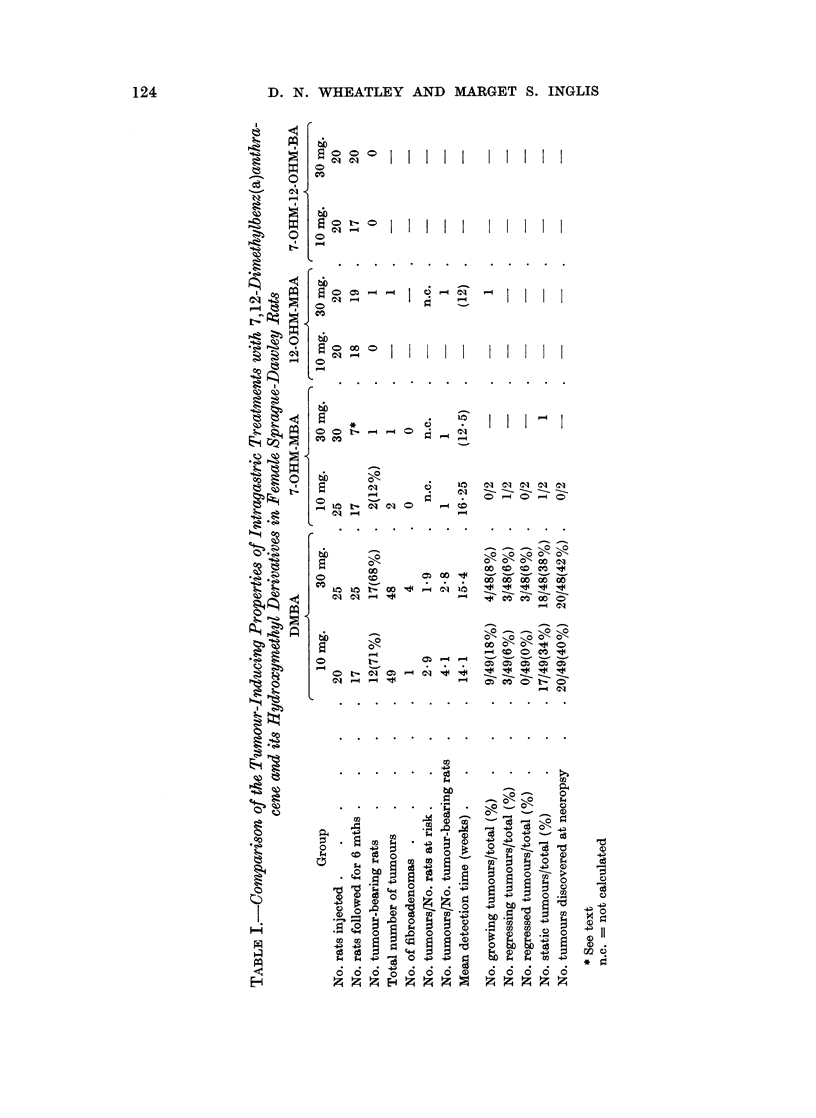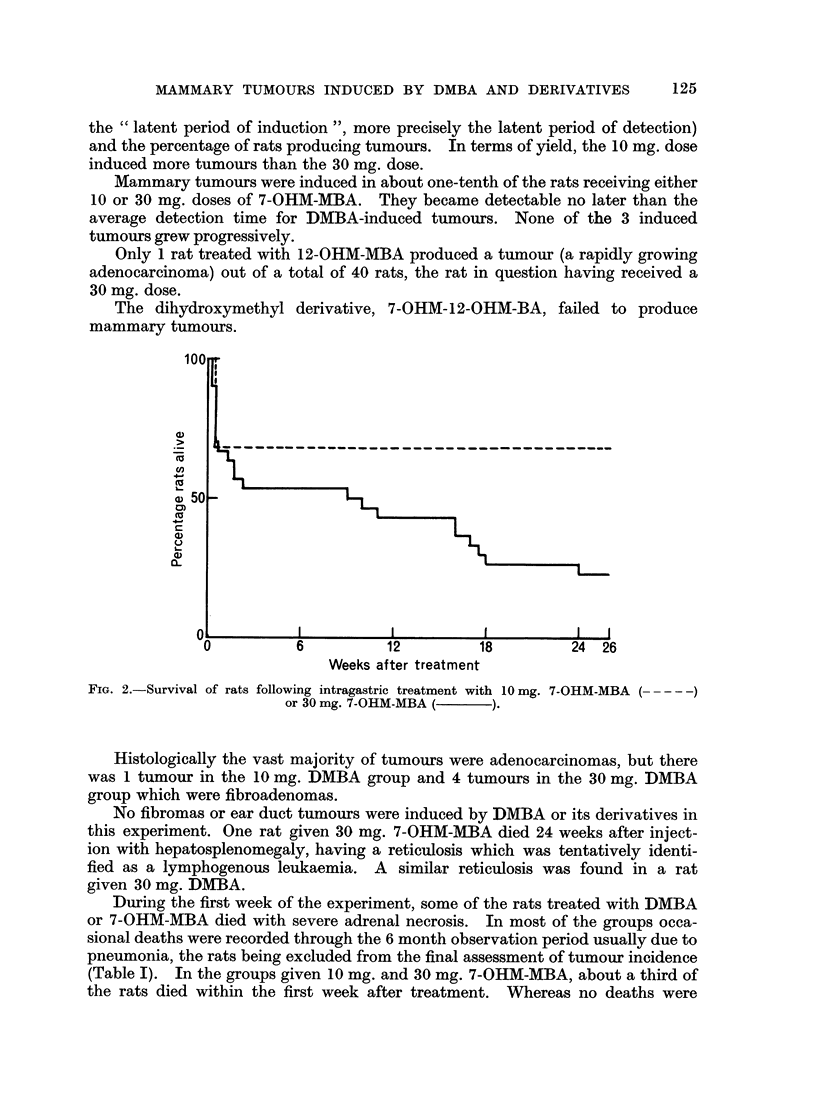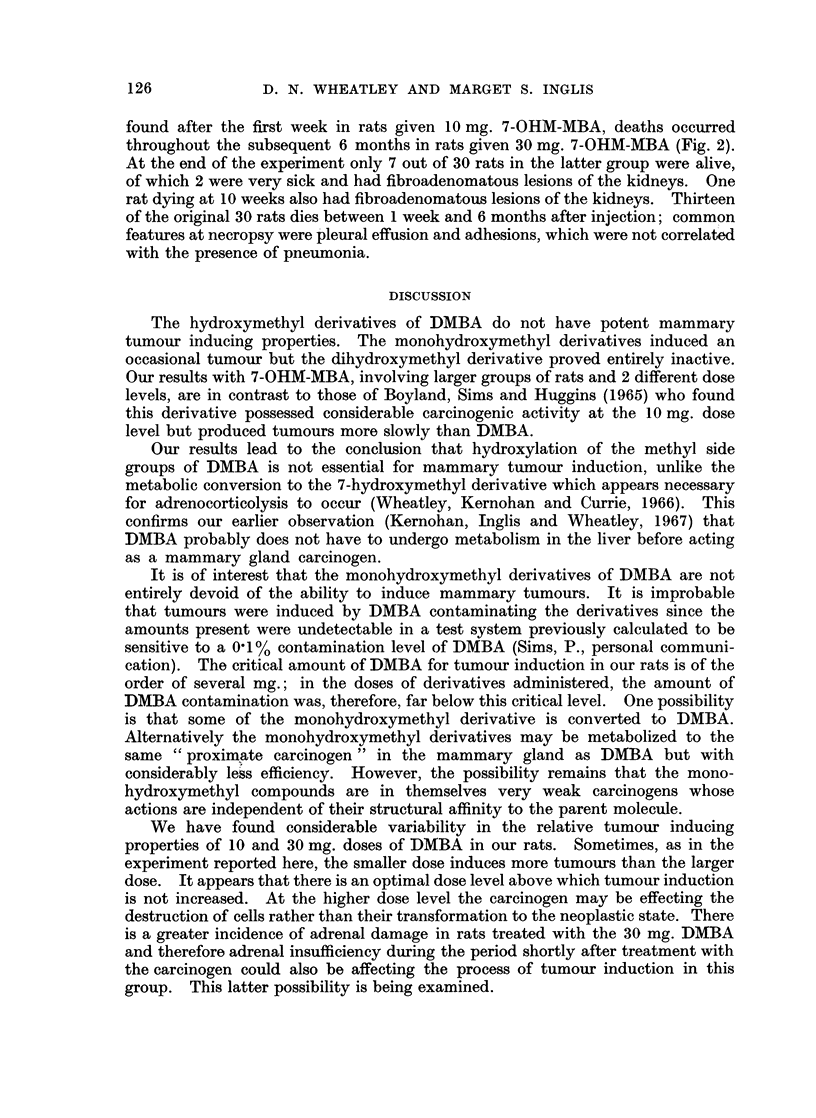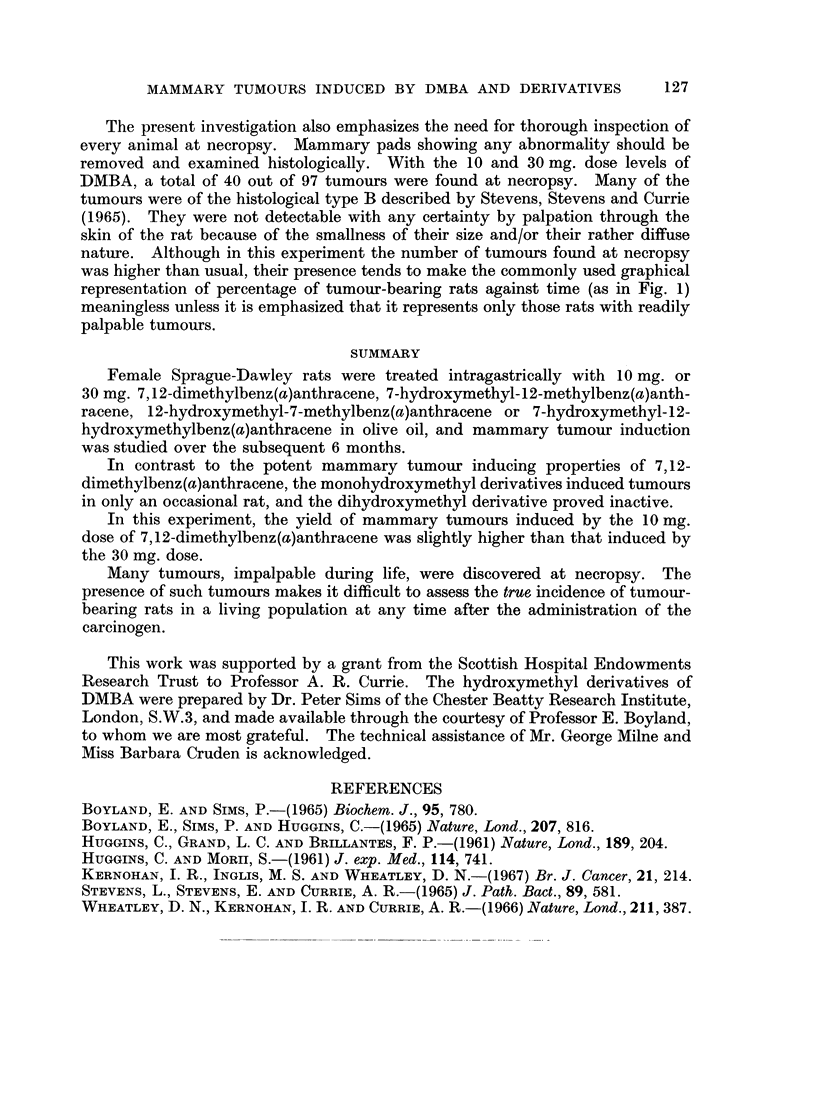# Mammary tumours induced in Sprague-Dawley female ats by 7,12-dimethylbenz(a)anthracene and its hydroxymethyl derivatives.

**DOI:** 10.1038/bjc.1968.17

**Published:** 1968-03

**Authors:** D. N. Wheatley, M. S. Inglis


### MAMMARY TUMOURS INDUCED IN SPRAGUE-DAWLEY FEMALE RATS BY 7,12-DIMETHYLBENZ(*a*)ANTHRACENE AND ITS HYDROXYMETHYL DERIVATIVES

D. N. WHEATLEY AND MARGET S. INGLIS

*From the Department of Pathology, University Medical Buildings, Foresterhill, Aberdeen*

Received for publication December 11, 1967

Administration of a single large dose of 7,12-dimethylbenz(*a*)anthracene (9,10-dimethyl-1,2-benzanthracene, DMBA) to Sprague-Dawley rats leads to the development of mammary tumours within 6–8 weeks (Huggins, Grand and Brillantes, 1961). It also results in the destruction of the inner zones of the adrenal cortex within 2–3 days (Huggins and Morii, 1961) but this occurs only after DMBA has been metabolized by the liver (Wheatley, Kernohan and Currie, 1966). Impairment of liver function by $CCl_4$ or partial hepatectomy, whilst preventing the adrenocorticolytic effect of DMBA injection, fails to influence mammary tumour induction significantly (Kernohan, Inglis and Wheatley, 1967). It is considered improbable, therefore, that mammary tumours are induced by a metabolite of DMBA produced in the liver.

DMBA is metabolized in the rat liver mainly by hydroxylation of its methyl side groups, unlike many other polycyclic hydrocarbons which undergo ring hydroxylation (Boyland and Sims, 1965). Hydroxymethyl derivatives—7-hydroxymethyl-12-methylbenz(*a*)anthracene (7-OHM-MBA), 12-hydroxymethyl-7-methylbenz(*a*)anthracene (12-OHM-MBA) and 7-hydroxymethyl-12-hydroxymethylbenz(*a*)anthracene (7-OHM-12-OHM-BA)—have been identified as products of DMBA after incubation with rat liver homogenates. Their ability to induce mammary tumours was investigated by Boyland, Sims and Huggins (1965) who found that the 7-hydroxymethyl derivative was carcinogenic but slower in producing tumours than DMBA whereas the 12-hydroxymethyl derivative was inactive. The results of our investigations with these derivatives form the basis of this report.

### MATERIALS AND METHODS

Sprague-Dawley female rats from an accredited stock were obtained from Oxford Laboratory Animal Colonies, Bicester, Oxon, England. They were treated with polycyclic compounds at 50 days of age when weighing about 150 g. They were fed modified Thompson rat cube containing 14 % skimmed milk (North-Eastern Agricultural Co-operative Society Ltd., Aberdeen, Scotland) and allowed water *ad libitum*. They were kept in groups of 5 rats per cage at a room temperature of 21–22° C. (70–72° F.) with natural daylight hours.

7,12-dimethylbenz(*a*)anthracene (DMBA), 7-hydroxymethyl-12-methylbenz(*a*) anthracene (7-OHM-MBA), 12-hydroxymethyl-7-methylbenz(*a*)anthracene (12-OHM-MBA) and 7-hydroxymethyl-12-hydroxymethylbenz(*a*)anthracene (7-OHM-12-OHM-BA) were dissolved in olive oil at concentrations of 10 and 15 mg./ml. In making up the compounds at these concentrations, the more hydrophilic derivatives remained to some extent in suspension rather than solution. The solutions were given to rats by stomach tube in doses of 1·0 ml. at the lower concentration (10 mg. dose) or 2·0 ml. at the higher concentration (30 mg. dose) after a 4-hour period of fasting.

Commencing 4 weeks after injection, rats were palpated each week for tumours. Tumours were measured and charted as described by Stevens, Stevens and Currie (1965). The animals were killed 6 months after injection and examined for tumours. Sections (5 $\mu$) stained with haematoxylin and eosin were prepared from every tumour or suspected tumour after fixation in 4% neutral buffered formaldehyde and paraffin wax embedding.

### RESULTS

Fig. 1 shows the percentage of rats with palpable tumours against time.

FIG. 1.—Percentage of rats bearing palpable mammary tumours against time after treatment of 50-day Sprague-Dawley female rats with DMBA and its hydroxymethyl derivatives. The inflexions towards the end of the curves for both 10 mg. and 30 mg. DMBA treatments introduce those rats with no palpable tumours which were found to have tumours at necropsy on week 26, so that the final figures attain the true percentage of tumour-bearing rats.

1. 10 mg. DMBA
2. 30 mg. DMBA
3. 10 mg. 7-OHM-MBA
4. 30 mg. 7-OHM-MBA
5. 10 mg. 12-OHM-MBA
6. 30 mg. 12-OHM-MBA
7. 10 mg. 7-OHM-12-OHM-BA
8. 30 mg. 7-OHM-12-OHM-BA

Table I gives an analysis of the induced tumours.

DMBA at 10 mg. and 30 mg. had comparable tumour inducing properties in our rats with respect to both time at which tumours became palpable (referred to as

TABLE I.—*Comparison of the Tumour-Inducing Properties of Intragastric Treatments with 7,12-Dimethylbenz(a)anthracene and its Hydroxymethyl Derivatives in Female Sprague-Dawley Rats*

| | DMBA | | 7-OHM-MBA | | 12-OHM-MBA | | 7-OHM-12-OHM-BA | |
|---|---|---|---|---|---|---|---|---|
| Group | 10 mg. | 30 mg. | 10 mg. | 30 mg. | 10 mg. | 30 mg. | 10 mg. | 30 mg. |
| No. rats injected | 20 | 25 | 25 | 30 | 20 | 20 | 20 | 20 |
| No. rats followed for 6 mths | 17 | 25 | 17 | 7* | 18 | 19 | 17 | 20 |
| No. tumour-bearing rats | 12(71%) | 17(68%) | 2(12%) | 1 | 0 | 1 | 0 | 0 |
| Total number of tumours | 49 | 48 | 2 | 1 | — | 1 | — | — |
| No. of fibroadenomas | 1 | 4 | 0 | 0 | — | — | — | — |
| No. tumours/No. rats at risk | 2·9 | 1·9 | n.c. | n.c. | — | n.c. | — | — |
| No. tumours/No. tumour-bearing rats | 4·1 | 2·8 | 1 | 1 | — | 1 | — | — |
| Mean detection time (weeks) | 14·1 | 15·4 | 16·25 | (12·5) | — | (12) | — | — |
| No. growing tumours/total (%) | 9/49(18%) | 4/48(8%) | 0/2 | — | — | 1 | — | — |
| No. regressing tumours/total (%) | 3/49(6%) | 3/48(6%) | 1/2 | — | — | — | — | — |
| No. regressed tumours/total (%) | 0/49(0%) | 3/48(6%) | 0/2 | — | — | — | — | — |
| No. static tumours/total (%) | 17/49(34%) | 18/48(38%) | 1/2 | 1 | — | — | — | — |
| No. tumours discovered at necropsy | 20/49(40%) | 20/48(42%) | 0/2 | — | — | — | — | — |

* See text n.c. = not calculated the " latent period of induction ", more precisely the latent period of detection) and the percentage of rats producing tumours. In terms of yield, the 10 mg. dose induced more tumours than the 30 mg. dose.

Mammary tumours were induced in about one-tenth of the rats receiving either 10 or 30 mg. doses of 7-OHM-MBA. They became detectable no later than the average detection time for DMBA-induced tumours. None of the 3 induced tumours grew progressively.

Only 1 rat treated with 12-OHM-MBA produced a tumour (a rapidly growing adenocarcinoma) out of a total of 40 rats, the rat in question having received a 30 mg. dose.

The dihydroxymethyl derivative, 7-OHM-12-OHM-BA, failed to produce mammary tumours.

FIG. 2.—Survival of rats following intragastric treatment with 10 mg. 7-OHM-MBA (– – – – –) or 30 mg. 7-OHM-MBA (————).

Histologically the vast majority of tumours were adenocarcinomas, but there was 1 tumour in the 10 mg. DMBA group and 4 tumours in the 30 mg. DMBA group which were fibroadenomas.

No fibromas or ear duct tumours were induced by DMBA or its derivatives in this experiment. One rat given 30 mg. 7-OHM-MBA died 24 weeks after injection with hepatosplenomegaly, having a reticulosis which was tentatively identified as a lymphogenous leukaemia. A similar reticulosis was found in a rat given 30 mg. DMBA.

During the first week of the experiment, some of the rats treated with DMBA or 7-OHM-MBA died with severe adrenal necrosis. In most of the groups occasional deaths were recorded through the 6 month observation period usually due to pneumonia, the rats being excluded from the final assessment of tumour incidence (Table I). In the groups given 10 mg. and 30 mg. 7-OHM-MBA, about a third of the rats died within the first week after treatment. Whereas no deaths were found after the first week in rats given 10 mg. 7-OHM-MBA, deaths occurred throughout the subsequent 6 months in rats given 30 mg. 7-OHM-MBA (Fig. 2). At the end of the experiment only 7 out of 30 rats in the latter group were alive, of which 2 were very sick and had fibroadenomatous lesions of the kidneys. One rat dying at 10 weeks also had fibroadenomatous lesions of the kidneys. Thirteen of the original 30 rats dies between 1 week and 6 months after injection; common features at necropsy were pleural effusion and adhesions, which were not correlated with the presence of pneumonia.

### DISCUSSION

The hydroxymethyl derivatives of DMBA do not have potent mammary tumour inducing properties. The monohydroxymethyl derivatives induced an occasional tumour but the dihydroxymethyl derivative proved entirely inactive. Our results with 7-OHM-MBA, involving larger groups of rats and 2 different dose levels, are in contrast to those of Boyland, Sims and Huggins (1965) who found this derivative possessed considerable carcinogenic activity at the 10 mg. dose level but produced tumours more slowly than DMBA.

Our results lead to the conclusion that hydroxylation of the methyl side groups of DMBA is not essential for mammary tumour induction, unlike the metabolic conversion to the 7-hydroxymethyl derivative which appears necessary for adrenocorticolysis to occur (Wheatley, Kernohan and Currie, 1966). This confirms our earlier observation (Kernohan, Inglis and Wheatley, 1967) that DMBA probably does not have to undergo metabolism in the liver before acting as a mammary gland carcinogen.

It is of interest that the monohydroxymethyl derivatives of DMBA are not entirely devoid of the ability to induce mammary tumours. It is improbable that tumours were induced by DMBA contaminating the derivatives since the amounts present were undetectable in a test system previously calculated to be sensitive to a 0·1% contamination level of DMBA (Sims, P., personal communication). The critical amount of DMBA for tumour induction in our rats is of the order of several mg.; in the doses of derivatives administered, the amount of DMBA contamination was, therefore, far below this critical level. One possibility is that some of the monohydroxymethyl derivative is converted to DMBA. Alternatively the monohydroxymethyl derivatives may be metabolized to the same "proximate carcinogen" in the mammary gland as DMBA but with considerably less efficiency. However, the possibility remains that the monohydroxymethyl compounds are in themselves very weak carcinogens whose actions are independent of their structural affinity to the parent molecule.

We have found considerable variability in the relative tumour inducing properties of 10 and 30 mg. doses of DMBA in our rats. Sometimes, as in the experiment reported here, the smaller dose induces more tumours than the larger dose. It appears that there is an optimal dose level above which tumour induction is not increased. At the higher dose level the carcinogen may be effecting the destruction of cells rather than their transformation to the neoplastic state. There is a greater incidence of adrenal damage in rats treated with the 30 mg. DMBA and therefore adrenal insufficiency during the period shortly after treatment with the carcinogen could also be affecting the process of tumour induction in this group. This latter possibility is being examined.

The present investigation also emphasizes the need for thorough inspection of every animal at necropsy. Mammary pads showing any abnormality should be removed and examined histologically. With the 10 and 30 mg. dose levels of DMBA, a total of 40 out of 97 tumours were found at necropsy. Many of the tumours were of the histological type B described by Stevens, Stevens and Currie (1965). They were not detectable with any certainty by palpation through the skin of the rat because of the smallness of their size and/or their rather diffuse nature. Although in this experiment the number of tumours found at necropsy was higher than usual, their presence tends to make the commonly used graphical representation of percentage of tumour-bearing rats against time (as in Fig. 1) meaningless unless it is emphasized that it represents only those rats with readily palpable tumours.

### SUMMARY

Female Sprague-Dawley rats were treated intragastrically with 10 mg. or 30 mg. 7,12-dimethylbenz(*a*)anthracene, 7-hydroxymethyl-12-methylbenz(*a*)anthracene, 12-hydroxymethyl-7-methylbenz(*a*)anthracene or 7-hydroxymethyl-12-hydroxymethylbenz(*a*)anthracene in olive oil, and mammary tumour induction was studied over the subsequent 6 months.

In contrast to the potent mammary tumour inducing properties of 7,12-dimethylbenz(*a*)anthracene, the monohydroxymethyl derivatives induced tumours in only an occasional rat, and the dihydroxymethyl derivative proved inactive.

In this experiment, the yield of mammary tumours induced by the 10 mg. dose of 7,12-dimethylbenz(*a*)anthracene was slightly higher than that induced by the 30 mg. dose.

Many tumours, impalpable during life, were discovered at necropsy. The presence of such tumours makes it difficult to assess the *true* incidence of tumour-bearing rats in a living population at any time after the administration of the carcinogen.

This work was supported by a grant from the Scottish Hospital Endowments Research Trust to Professor A. R. Currie. The hydroxymethyl derivatives of DMBA were prepared by Dr. Peter Sims of the Chester Beatty Research Institute, London, S.W.3, and made available through the courtesy of Professor E. Boyland, to whom we are most grateful. The technical assistance of Mr. George Milne and Miss Barbara Cruden is acknowledged.

### REFERENCES

BOYLAND, E. AND SIMS, P.—(1965) *Biochem. J.*, **95**, 780.
BOYLAND, E., SIMS, P. AND HUGGINS, C.—(1965) *Nature, Lond.*, **207**, 816.
HUGGINS, C., GRAND, L. C. AND BRILLANTES, F. P.—(1961) *Nature, Lond.*, **189**, 204.
HUGGINS, C. AND MORII, S.—(1961) *J. exp. Med.*, **114**, 741.
KERNOHAN, I. R., INGLIS, M. S. AND WHEATLEY, D. N.—(1967) *Br. J. Cancer*, **21**, 214.
STEVENS, L., STEVENS, E. AND CURRIE, A. R.—(1965) *J. Path. Bact.*, **89**, 581.
WHEATLEY, D. N., KERNOHAN, I. R. AND CURRIE, A. R.—(1966) *Nature, Lond.*, **211**, 387.